# Effect of preemptive dexamethasone and etoricoxib on postoperative period following impacted third molar surgery - a randomized clinical trial

**DOI:** 10.4317/medoral.23095

**Published:** 2019-10-27

**Authors:** Éwerton Daniel Rocha Rodrigues, Gabriela Soares Pereira, Belmiro Cavalcanti do Egito Vasconcelos, Renato da Costa Ribeiro

**Affiliations:** 1 Post-graduate student, Departmant of Oral and Maxillofacial Surgery, School of Dentistry, Universidade de Pernambuco, Camaragibe, PE, Brazil; 2 Doctor of Dental Surgery, Universidade Federal do Piauí, Teresina, PI, Brazil; 3 Assistant Professor, Departmant of Oral and Maxillofacial Surgery, School of Dentistry, Universidade de Pernambuco , Camaragibe, PE, Brazil; 4 Oral and Maxillofacial Surgeon, University Hospital, Universidade Federal do Piauí, Teresina, PI, Brazil

## Abstract

**Background:**

The aim of this study was to compare the anti-inflammatory effects of dexamethasone and etoricoxib after third molar extraction.

**Material and Methods:**

A prospective, randomized, controlled, split-mouth study was conducted. 19 volunteers were allocated randomly to receive 90mg etoricoxib 1 hour prior to the procedure or 4mg intramuscular dexamethasone immediately after anesthesia. Baseline measurements were obtained preoperatively, and subsequent assessments were made on immediate postoperative, at 72 hours and 7 days after surgery to measure postoperative facial swelling by use of linear measurements, interincisal mouth opening width and visual analog scale score for pain. The amount of analgesics consumed was recorded. Descriptive statistics and the independent-samples t-test were used to compare the two groups at * P * < 0.05.

**Results:**

Dexamethasone was effective in the control roasted edema for measurements of the mandibular angle - wing of the nose and mandibular angle - labial commissure 72 hours after surgery. And for the measurement mandibular angle - mentum, in the time of 72 hours and 7 days. There was no statistically significant difference in relation to pain and trismus.

**Conclusions:**

Considering significant results for some measures of the variable edema for the group that used intramuscular dexamethasone and the difference without statistical significance between groups for the other variables studied, we seem to reflect the intramuscular indication of the corticosteroid in a single dosage in relation to the use of etoricoxib as pre-emptive medication.

** Key words:**Corticosteroids, COX-2 selective, third-molar surgery.

## Introduction

The extraction of mandibular third molars is the most frequent intervention in oral surgery (1,2). Although performed using a meticulous surgical technique, the trauma resulting from this surgery leads to an acute inflammatory reaction with considerable pain, swelling and trismus (3), which affects quality of life by limiting the capacity to perform activities of daily living, especially in the first three days after surgery (4). Preemptive analgesia has been employed for the management of these postoperative symptoms and consists of the pharmacological modulation of local and systemic mediators of pain and inflammation to inhibit the nociceptive stimulation resulting from this surgical procedure (4,5). Different groups of medication have been employed to reduce the postoperative inflammatory response, such as steroidal and non-steroidal anti-inflammatory drugs (SAIDs and NSAIDs) (1,4-6).

SAIDs have been used for decades to control inflammation following third molar surgey (3) by inhibiting the enzyme phospholipase A2, which is a chemical mediator responsible for the induction of arachidonic acid. This action results in the reduction of proinflammatory mediators, such as prostaglandins, prostacyclins, leukotrienes and thromboxane A2 (1,6). Dexamethasone is one of the most widely used SAIDs in third molar surgery due to its predominant glucocorticoid effect, minimal sodium retention activity and long half life (3). Enteral administration is considered convenient and safe, but requires the patient’s cooperation and there may be a change in the biological response due to the pharmacokinetics of the drug; moreover, there is a late onset effect in comparison to intramuscular administration (7). The injection of corticosteroids in the medial pterygoid muscle is accepTable, since this is an area that is anesthetized during the operation and this method eliminates the possibility of gastrointestinal side effects (7).

Another method for preemptive analgesia is the use of NSAIDs, which reduce the synthesis of prostaglandins derived from arachidonic acid through the inhibition of the enzyme cyclooxygenase (COX). Etoricoxib is a selective COX-2 inhibitor that has an analgesic effect for the treatment of acute pain, with a fast onset and half life of approximately 25 hours (1,8). COX-2-selective NSAIDs are associated with fewer gastrointestinal side effects, do not affect platelet function and are well tolerated. In a study comparing etoricoxib, ibuprofen and paracetamol (acetaminophen) combined with codeine and placebo, Brown *et al*. (9) concluded that pain control was more effective with etoricoxib and ibuprofen and that fewer patients in the etoricoxib group required rescue medication.

Although previous studies have compared corticosteroids and NSAIDs for the control of the symptoms of inflammation following third molar surgery, no studies have been published comparing dexamethasone administered intramuscularly to etoricoxib administered orally. Perhaps analgesic efficacies of etoricoxib administered before extraction of impacted lower third molar may be more effective than injection of dexamethasone. Therefore, the aim of the present study was to compare the anti-inflammatory effects of dexamethasone administered to the medial pterygoid muscle and etoricoxib administered orally following third molar extractions.

Materials and Methods

- Study design

A randomized, double-blind, clinical trial with a split-mouth design was conducted. The experimental part was performed at the university hospital of the Universidade Federal do *Pi*auí. This study received approval from the human research ethics committee of the study institution (certificate number: 67695817.6.0000.8050).

The inclusion criteria were 1) age 18 to 35 years, 2) absence of systemic disease (ASA I), 3) no use of medication in the previous seven days, 4) mandibular third molars in similar positions with similar root formation patterns, 5) absence of allergy to the drugs used in the study and 6) surgical site with no current signs or symptoms of infection. Patients who met any of the following criteria were excluded from the study: 1) pregnancy or lactation; 2) history of gastrointestinal bleeding or peptic ulcer; 3) allergy to aspirin or NSAIDs; 4) liver, kidney, blood or central nervous system disease; 5) continued use of psychoactive drugs, analgesics, SIADs or NSAIDs; and (6) current smoking habit.

Patients were scheduled for surgery in two separate clinical sessions (one side at a time) at least 15 days apart. Subjects were allocated to one of two groups through a computer-generated randomization code (Microsoft Excel) according to the medication received (Group A – preoperative administration of 4 mg of dexamethasone (Aché, Brazil) in the medial pterygoid muscle immediately after the administration of anesthesia; Group B – preoperative oral administration of 90 mg of etoricoxib (DSM, Brazil) one hour prior to surgery). Information on the type of medication provided to each study subject was withheld from the patient, clinical investigator (responsible for patient follow-up examinations and outcome measurements), and statistician. Prior to surgery, a list containing a randomized distribution of all surgical sites and pain medications to be administered was held in a sealed envelope by an external study collaborator, who was unaware of the study protocol and had no further participation in this clinical trial other than to guarantee a study design.

To ensure blinding, 1 ml of saline solution was injected intramuscularly during the procedure in which etoricoxib was used and a placebo pill was administered one hour prior to surgery during the procedure in which dexamethasone was used. All patients received 12 pills of paracetamol 750 mg as the rescue drug to be used as deemed necessary for the control of postoperative pain and were instructed not to exceed a total of four pills in a 24-h period.

- Calculation of sample size

A pilot study was conducted with eight patients (16 mandibular third molars) to assist in the calculation of the sample size for the main study to enable the statistical rejection of the null hypothesis with an 80% power and 95% confidence interval. The maximum reasonable difference between the mean obtained from the sample and the true mean of the population was 13. Considering an α error = 0.05 and seven degrees of freedom a sample of 19 patients was determined.

- Surgical procedure

The surgical procedures were performed by a single surgeon using a standardized technique in an outpatient setting and all members of the surgical team followed biosafety protocols. The same surgical protocol was used for both sides to reduce the difference in trauma. There was no standardization regarding which side would be operated first. The patients received local anesthesia with 2% mepivacaine and epinephrine 1:200.000. The muco-periosteal flap was detached, followed by ostectomy and tooth sectioning under irrigation with saline solution. After smoothing the bone edges, the surgical wound was irrigated abundantly with 0.9% saline solution and the suture was performed with nylon 4-0 thread (Ethicon).

- Assessment methods

**Figure 1:**
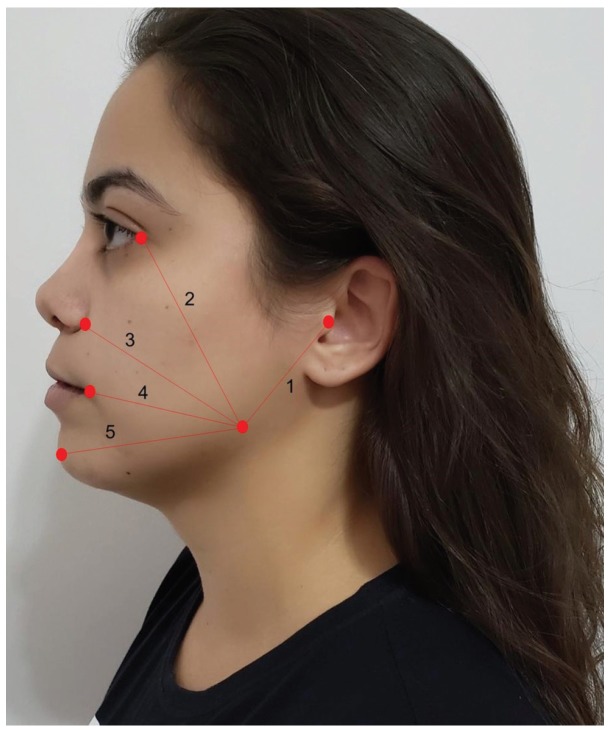
Measurement of swelling.

Postoperative facial swelling was assessed using five measurements described by Neupert *et al*., (10): 1- angle of the mandible/tragus, 2- angle of the mandible/corner of the eye; 3- angle of the mandible/ala of the nose; 4- angle of the mandible/lip commissue and 5- angle of the mandible/pogonion (Fig. 1). The preoperative values were compared to those determined immediately after surgery, 72 hours after surgery and seven days after surgery. Trismus was measured as the difference in maximal mouth opening. This procedure was repeated immediately after surgery, 72 hours after surgery and seven days after surgery.

Postoperative pain was determined using the Visual Analog Scale (VAS), 10-cm long, that ranged from 0 (absence of pain) to 10 (maximum pain) (3-5). The amount of the rescue drug used and time of surgery (from the beginning of the incision to the end of the suture) was also recorded.

- Data analysis 

Central tendency (mean and standard deviation) and dispersion (minimum and maximum) measures were used. For the quantitative variables, the Shapiro-Wilk test was used to determine adherence to normal distribution. The Student’s t-test for paired samples and Wilcoxon test were used for the comparisons of the groups (edema measurements, total and mean number of analgesics taken in postoperative period). Pearson’s correlation coefficients were calculated to determine correlations between parametric variables (correlation of pain with number of analgesics) and Spearman’s correlation coefficients were calculated to determine correlations between nonparametric variables (correlation of trismus with over time). The data were tabulated electronically using Microsoft Office Excel and analyzed with the aid of the IBM Statistical Package for the Social Sciences (SPSS version 20.0).

## Results

Nineteen individuals (11 women and eight men) were recruited between September 2017 and March 2018 and adhered to the study protocol. Age ranged from 18 to 32 years (mean: 23.73 years). No statistically significant differences in surgery time were found between the different treatments (Table 1).

**Table 1:**
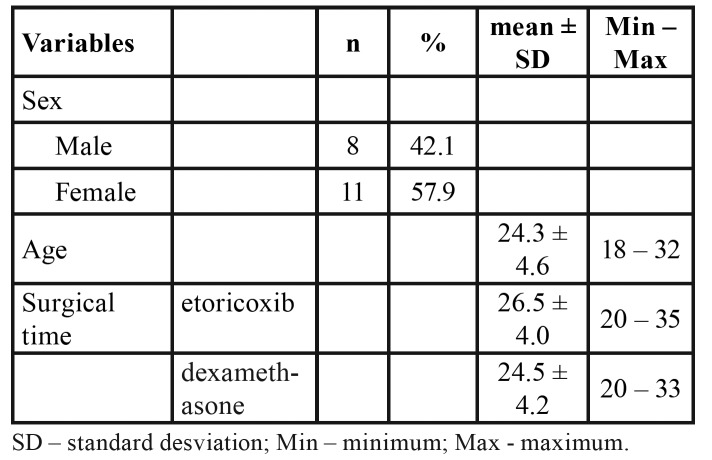
Characteristics of patients.

In the comparison of the edema between groups A and B, it was possible to observe that there was no statistically significant difference for 1 and 2 measurements between evaluation times (t-test; Wilcoxon test). Statistically significant differences were found in the 3 and 4 measurements between the preoperative evaluation and 72-hour evaluation. Significant differences for the 5 measurement were found at both the 72-hour and seven-day evaluations (Table 2).

**Table 2:**
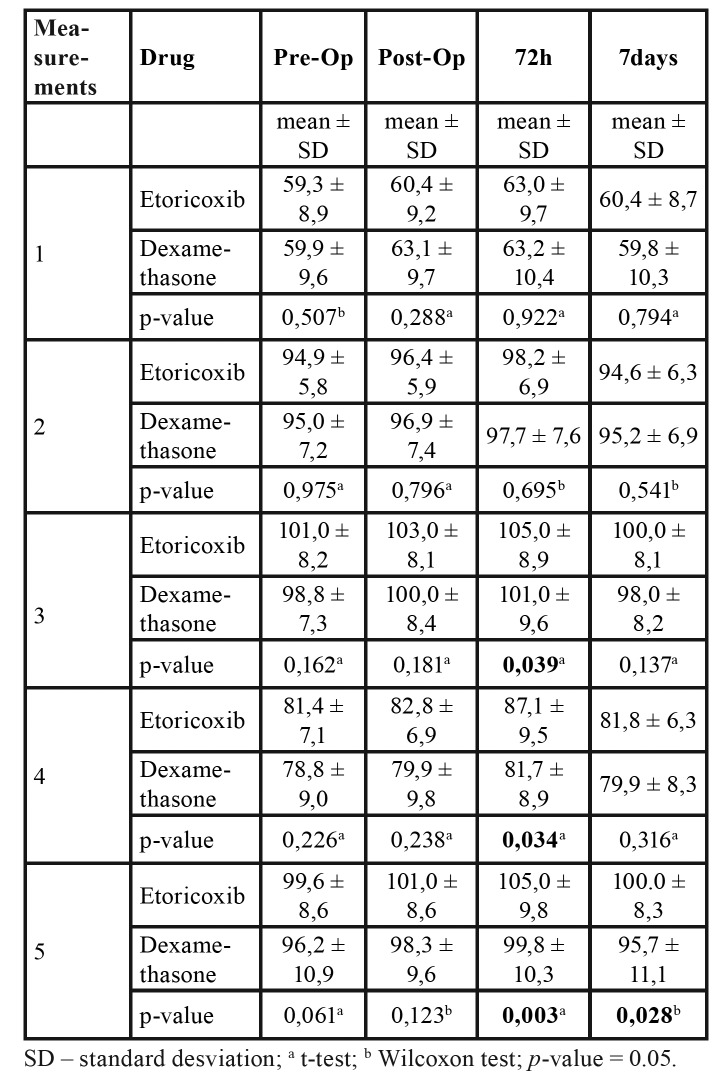
Facial measurements at pre-operative, immediate post-operative, 72-hour and seven-day evaluations.

**Figure 2:**
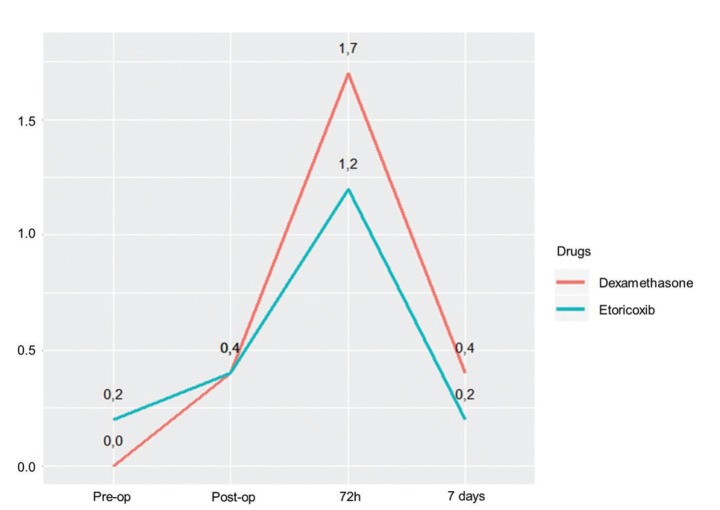
Mean pain intensity at pre-operative, immediate post-operative, 72-hour and seven-day evaluations.

**Figure 3:**
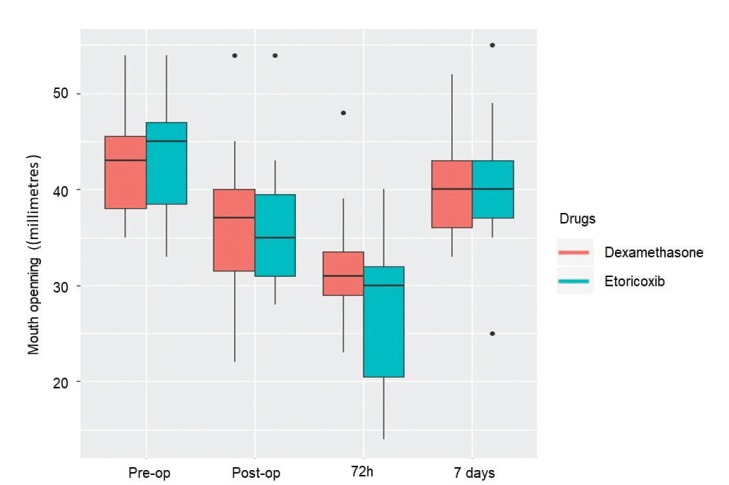
Boxplot of trismus at pre-operative, immediate post-operative, 72-hour and seven-day evaluations.

Mean postoperative pain was higher in the dexamethasone group, but the difference was not statistically significant (Fig. 2). Trismus 72 hours after surgery was worse in the etoricoxib group, but the difference did not achieve statistical significance (Fig. 3). No significant difference between groups was found in the number of rescue drugs taken (Table 3). A positive correlation was found between pain intensity and number of rescue drugs taken (*p* < 0.05) (Table 4).

**Table 3:**
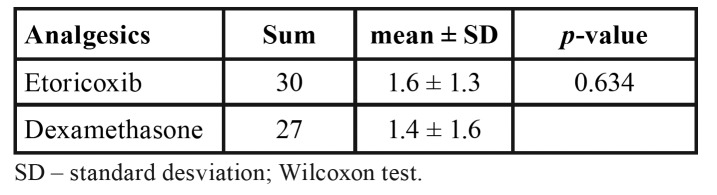
Total and mean number of analgesics taken in postoperative period.

**Table 4:**
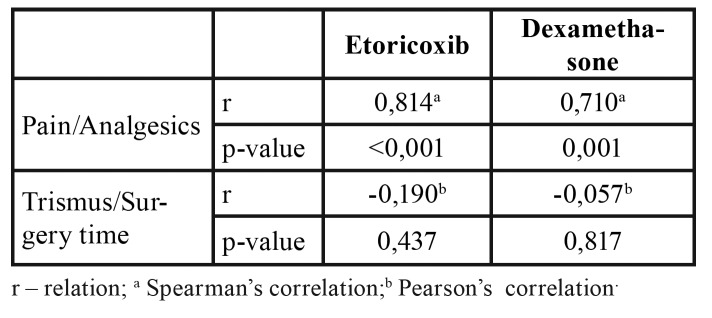
Correlation matrix of pain with number of analgesics and trismus over time.

## Discussion

Excessive swelling, pain and trismus are often associated with this type of surgery, (3,11,12) the intensity of which varies with the extent of the trauma, which is directly related to the degree of surgical difficulty. The dental pain model used after third molar surgeries is one of the most common and widely accepted for the evaluation of analgesics in humans (1,13). Anti-inflammatory agents have been used preemptively to reduce the inflammatory signs and symptoms resulting from this surgical procedure (4,5). The present study evaluated the effectiveness of an NSAID administered orally and a corticosteroid administered intramuscularly, which are widely used classes of drugs in third molar extractions (4,14).

Levels of postoperative pain depend on the tissue trauma caused by the surgical procedure (2-5,15-17). In a previous study, mean pain scores were lower in the group that made use of dexamethasone in comparison to the control group at all evaluation times (2). However, there is no consensus on the role of corticosteroids in the control of postoperative pain. While some researchers state that corticosteroids alone have no significant analgesic effect, (6,18) others found that the use of SAIDs led to reductions in mean pain following third molar extractions (2,3,11). In a clinical trial conducted by Costa *et al*. (4), peak postoperative pain occurred six hours after the procedure in patients medicated with etoricoxib and mean postoperative pain scores were significantly lower in these patients. One of the limitations of the present study was the cooperation of the patients in filling out the VAS charts, which compromised the assessment of pain in the first 48 hours after surgery. Therefore, the analysis of this variable was only performed on the third day, when the patients returned for the evaluations of swelling and trismus. Lower mean pain scores were found when the patients took etoricoxib, but the difference compared to dexamethasone was non-significant.

In the present study, the method described by Neupert *et al*. (13) was used. Costa *et al*. (4) analyzed the preemptive effect of etoricoxib (120 mg) and placebo on inflammatory events after the removal of third molars and found no significant difference in facial measurements between groups at any evaluation time. Sotto-Maior *et al*. (1) compared the anti-inflammatory effects of etoricoxib (120 mg) and dexamethasone (4 mg) administered orally one hour prior to the procedure and found no significant differences in postoperative swelling. In contrast, dexamethasone was more effective at controlling edema regarding the 3 and 4 measurements after 72 hours as well as the 5 measurement after 72 hours and seven days in the present investigation. Mojsa *et al*. (19) evaluated the submucosal injection of dexamethasone and found that peak swelling in patients who received placebo occurred on the third day and these patients had significantly larger facial measurements. Antunes *et al*. (20) compared the administration of dexamethasone intramuscularly (masseter muscle), orally and a placebo and found that the control group had the greatest swelling. Moreover, the patients who received oral dexamethasone had greater swelling values in comparison to those who received the medication intramuscularly, but the difference did not achieved statistical significance. These findings suggest that the parenteral administration of dexamethasone achieves better results due to its faster onset in comparison to enteral administration.

Moore *et al*. (21) found that trismus was more severe in all treatment groups on the first or second day after surgery and returned to normal beginning on the seventh day. Majid (22) compared the effects of dexamethasone administered through submucosal and intramuscular routes and found a greater occurrence of trismus in the first postoperative day, with no significant difference between the patients submitted to different the administration routes. Similarly to the findings cited above, no significant different in trismus was found between the groups in the present investigation. In a systematic review, Almeida *et al*. (23) found that the preemptive administration of corticosteroids achieved better results regarding the control of trismus, which may be explained by the fact that the drug is made available to the organism prior to the tissue injury. According to Alexander and Throndson (18), to obtain the maximum expected benefit from the preemptive use of corticosteroids, administration should be performed two to four hours prior to the procedure to obtain adequate tissue levels.

The use of rescue medication adds an additional variable to the research design. da Costa Araújo *et al*. (12) state that the use of analgesics in the postoperative period is the most difficult aspect to control in a study due to the difficulty in establishing a standardized method that guides the use of these medications on the part of patients and may lead to overestimation of the beneficial effect of the group that took more rescue medication, increased risk of bias. In an attempt to compensate for this risk of bias, a comparison was made of the amount of rescue medication used in both groups. No statistically significant difference was found in the mean number of analgesics used in each group (1.6 in the etoricoxib group and 1.4 in the dexamethasone). The present findings differ from those described by Costa *et al*. (4), who found that the mean number of capsules of the rescue drug consumed in the first 24 hours and total amount rescue drug consumed was significantly lower in the group that received etoricoxib, which led to a significant reduction in postoperative pain and the need for the rescue drug. Sotto-Maior *et al*. (1) compared the anti-inflammatory effects of etoricoxib (120 mg) and dexamethasone (4 mg) administered one hour prior to the procedure and found no statistically significant difference in the amount of rescue drug used, which is similar to the findings described in the present study.

The drugs evaluated (dexamethasone and etoricoxib) in the respective doses and administration routes achieved similar effectiveness with regard to controlling pain and trismus following the extraction of mandibular third molars. Moreover, dexamethasone achieved better results with regard to the control of swelling for the 3 and 4 measurements at 72 hours as well as the 5 measurement at both 72 hours and seven days after surgery. Within the limitations of the study, the intramuscular administration of this corticoid in a single dose is suggested rather than the use of etoricoxib as a preemptive strategy in third molar surgeries.
